# Blockade of phospholipid scramblase 1 with its N-terminal domain antibody reduces tumorigenesis of colorectal carcinomas *in vitro* and *in vivo*

**DOI:** 10.1186/1479-5876-10-254

**Published:** 2012-12-24

**Authors:** Chung-Wei Fan, Chun-Yu Chen, Kuei-Tien Chen, Chia-Rui Shen, Yung-Bin Kuo, Ya-Shan Chen, Yeh-Pin Chou, Wei-Shan Wei, Err-Cheng Chan

**Affiliations:** 1 Department of Colorectal Surgery, Chang Gung Memorial Hospital, Keelung, Taiwan; 2 College of Medicine, Chang Gung University, Taoyuan, Taiwan; 3 Graduate Institute of Biomedical Sciences, Chang Gung University, Taoyuan, Taiwan; 4 Department of Medical Biotechnology and Laboratory Science, Chang Gung University, Taoyuan, Taiwan; 5 Division of Hepato-Gastroenterology, Chang Gung Memorial Hospital, Kaohsiung Medical Center, Kaohsiung, Taiwan; 6 Graduate Institute of Biochemical and Biomedical Engineering, Chang Gung University, Taoyuan, Taiwan

**Keywords:** Phospholipid scramblase 1, Retinoblastoma dephosphorylation, Cyclin D1, Cell cycle G1/S arrest, Colorectal carcinomas

## Abstract

**Background:**

Membrane-bound phospholipid scramblase 1 (PLSCR1) is involved in both lipid trafficking and cell signaling. Previously, we showed that PLSCR1 is overexpressed in many colorectal carcinomas (CRCs). In the present study, we investigated the tumorigenic role of PLSCR1 in CRC and suggest that it is a potential therapeutic target.

**Methods:**

To identify PLSCR1 as a therapeutic target, we studied the tumorigenic properties of CRC cell lines treated with a monoclonal antibody (NP1) against the N-terminus of PLSCR1 *in vitro* and *in vivo*. We also investigated cell cycle status and epidermal growth factor receptor–related pathways and downstream effectors of PLSCR1 after blocking its function with NP1.

**Results:**

Treating CRC cells with NP1 *in vitro* and *in vivo* decreased cell proliferation, anchorage-independent growth, migration, and invasion. Adding NP1 to the CRC cell line HT29 caused arrest at G1/S. Treating HT29 cells with NP1 significantly decreased the expression of cyclin D1 and phosphorylation levels of Src, the adaptor protein Shc, and Erks. The reduced level of cyclin D1 led to an increase in the activated form of the tumor suppressor retinoblastoma protein via dephosphorylation. These actions led to attenuation of tumorigenesis.

**Conclusions:**

Therefore, PLSCR1 may serve as a potential therapeutic target for CRC.

## Introduction

Previously, we identified several differentially expressed proteins in colorectal carcinoma (CRC) cells using gel-assisted digestion and label-free mass spectrophotometry [1]. Among the candidate proteins, we demonstrated a novel target, phospholipid scramblase1 (PLSCR1), which is overexpressed in most CRC tissues. Lower expression of PLSCR1 is associated with longer patient survival [2]. The molecular characteristics of PLSCR1 and the mechanisms by which it regulates tumorigenesis remain unclear. PLSCR1 is a member of the family of membrane proteins that have been proposed to mediate accelerated transbilayer movement of plasma membrane phospholipids under conditions of elevated cytoplasmic Ca^2+^[3,4]. In addition, PLSCR1 also seems to function as a signal transduction molecule. Despite progress in this area of PLSCR1-related biological function, with few exceptions little is known about the roles of PLSCR1 in the control of tumor cell proliferation or transformation. PLSCR1 interacts with onzin, which may promote apoptosis mediated by c-Myc [5]. In addition, the effects of PLSCR1 on cell proliferation and maturation may be related to altered expression of cellular inositol triphosphate receptors [6]. Granulocyte precursors derived from mice deficient in PLSCR1 show impaired proliferation and maturation in the presence of cytokines [7]. Moreover, ovarian carcinoma cell growth *in vivo* is suppressed by interferon-inducible PLSCR1 [8]. PLSCR1 has been reported to be a substrate for protein kinase Cδ, thereby elevating phosphatidylserine exposure in cells undergoing apoptosis [9]. PLSCR1 is also a substrate of a tyrosine kinase associated with the IgE receptor [10], c-Abl tyrosine kinases [11], and c-Src [12], implying a possible role for PLSCR1 in one or more intracellular signaling pathways that regulate cell proliferation or apoptosis.

PLSCR1 regulates cell transformation and proliferation upon growth factor stimulation. This process is initiated through the phosphorylation of membrane-bound PLSCR1 at Tyr^60^ and Tyr^74^ by Src kinase. PLSCR1 consequently interacts with both the Shc adaptor protein and the epidermal growth factor receptor (EGFR) in stimulated cells [12]. Both the phosphorylation sites and the Shc and Src binding sites are located in the N-terminal domain of PLSCR1, suggesting that this peptide domain may be a potential target in cancer therapy.

We suggest that preventing phosphorylation and binding of PLSCR1 may impair cell proliferation and transformation in the presence of growth factors. For this purpose, we prepared a monoclonal antibody, NP1, against the N-terminal domain of PLSCR1. We show that NP1 significantly attenuates the tumorigenic properties of CRC cell lines *in vitro* and *in vivo*.

## Materials and methods

### Cell culture

The HT29, HCT15, HCT116, CoLo205, LoVo, and WiDr CRC cell lines were maintained in RPMI-1640 medium (Invitrogen, Carlsbad, CA) supplemented with 10% fetal bovine serum (FBS) and antibiotics at 37°C in 5% CO_2_. SW480 and SW620 CRC cell lines and Hep G2 hepatocellular carcinoma cells were grown in Dulbecco’s modified Eagle medium (Invitrogen) with 10% FBS plus antibiotics at 37°C in 5% CO_2_. WI-38 human fibroblasts were cultured in Modified Eagle Medium (Invitrogen) containing 10% FBS, 2 mM l-glutamine, 1 mM sodium pyruvate, and antibiotics at 37°C in 5% CO_2_. For experiments, cells were passaged one day before treatment.

### Antibodies and reagents

Mouse IgG_1_ (MAB002) was purchased from R&D Systems (Minneapolis, MN). Antibodies against human leukocyte antigen (HLA) Class I (W6/32), cyclin D1 (EPR2241IHC), retinoblastoma 1 (1F8), and anti-phospho retinoblastoma 1 (Ser780) (E182) were purchased from Novus Biologicals (Littleton, CO). Mouse anti-human CD47 was purchased from BD Biosciences (San Jose, CA). Alexa Fluor® 488–conjugated goat anti-mouse IgG was purchased from Invitrogen (Taipei, Taiwan). Antibodies against EGFR (#2232), Src (mouse monoclonal L4A1), p44/42 MAPK (Erk1/2; monoclonal 137F5), phospho-Src family (Tyr416; rabbit monoclonal 100F9), phospho-Shc (Tyr239/240), phospho-Shc (Tyr317; #2431), and phospho-p44/42 MAPK (Erk1/2; Thr202/Tyr204; mouse monoclonal, E10) were purchased from Cell Signaling (Danvers, MA). Rabbit anti-Shc polyclonal IgG (06–203) was purchased from Millipore (Temecula, CA).

### Generation of monoclonal antibody NP1

NP1 against PLSCR1 was produced in mice using the peptide DKQNSQMNASHPETNLPVGYPPQYPPTAF(C), which corresponds to residues 2–30 of human PLSCR1 [UniProt: O15162]. The sequence of this peptide contains multiple proline-rich domain, like PXXP and PPXY, and is specific to human PLSCR1. Antibodies were produced and affinity purified according to previously described procedures [13]. Briefly, a six week-old BALB/c mouse was subcutaneously immunized with PLSCR1 peptide (400 μg) emulsified in Freund’s complete adjuvant (Wako chemical, Richmond, VA). Immunization were repeated at 7-day intervals in incomplete Freund’s adjuvant (3 times). Spleen cells prepared from mouse immunized with the peptides were fused with Sp2/o-Ag14 mouse myeloma cells (1:1 ratio). Hybridoma specificity was tested with an enzyme-linked immunosorbent assay (ELISA) comparing bovine serum albumin (control) and the PLSCR1-specific peptide. All specific positive clones were then tested with western blotting against CRC cell lysates. One hybridoma (designated as NP1, IgG_1_) was obtained after subcloning by limiting dilution.

### Western blot analysis

Proteins were extracted from cells according to routine procedures and quantified with the DC™ protein assay method (Bio-Rad, Hercules, CA). Proteins were separated with SDS-PAGE. The blots were incubated with antibodies, developed with enhanced chemiluminescence reagents and exposed to Kodak Biomax light film. The immunoblot images were acquired by Imagemaster (Amersham Pharmacia, Piscataway, NJ). The intensity of each band was quantified by densitometry and analyzed with Multi Gauge Version 3.0 software (Fuji PhotoFilm, Tokyo, Japan).

### Immunohistochemical analysis of the tissue array

Tissue array specimens of normal and tumor organ tissues were purchased from Biomax (FDA807, Rockville, MD). Following the manufacturer’s protocol, immunohistochemistry was performed on tissue array specimens using a diaminobenzidine detection kit (Dako, AR155, Glostrup, Denmark). Antibody NP1 was diluted in 1 μg/mL for immunohistochemistry. PLSCR1 expression was categorized as positive or negative and was evaluated according to the simplified H scoring [14], which is based on the percent of cells stained (3, ≥90%; 2, 50–89%; 1, 10–49%; 0, 0–9%) and the intensity of cell staining on a 0–3 scale (3, strong; 2, moderate; 1, weak; 0, no staining). The two scores were multiplied by each other and divided by 3 to obtain the final score. Positive staining was defined as a final score ≥1.

### Cell proliferation assay

Cell proliferation was evaluated with the CellTiter 96® AQueous One Solution Reagent (Promega, Madison, WI). Cells were plated in 96-well plates (1 × 10^3^ cells/well) and cultured for various periods at 37°C. NP1 was used at final concentrations of 0–10 μg/mL. At the end of each incubation time, the medium in each well was replaced with fresh medium containing 20 μL CellTiter 96® AQueous One Solution Reagent, and the plate was incubated for 1 h. After incubation, the relative amounts of formazan were determined by measuring the absorbance at 490 nm using an ELISA reader (Fusion, Packard BioScience, Meriden, CT). For another experiment, we used a concentration of 10 μg/mL NP1 and evaluated growth until day 7 using the cell proliferation assay.

### Anchorage-independent transformation assay

Cells (6,000/well) in a 6-well plate were exposed to NP1 (20 μg/mL) in 1 mL agar (0.5%) containing 10% FBS. The cultures were incubated at 37°C in 5% CO_2_ for 14 days. The medium was changed two times per week. The 6-well plates were stained with 0.5 mL of 0.005% crystal violet for at least 1 h. Cell colonies were examined using a dissecting microscope.

### Wound healing assay

The wound healing assay was performed according to the manufacturer’s protocol (ibidi, Martinsried, Germany). Briefly, a culture insert (ibidi) was transferred to a well of a 6-well plate to generate a cell-free gap of about 500 μm. After cell attachment, the culture inserts were gently removed with sterile tweezers. The cells were incubated with medium supplemented with 1% FBS and treated with NP1 (20 μg/mL). Images of the cell-free gap were taken at exactly the same position on the cell culture plate using an inverted microscope (ECLIPSE TS100, Nikon, Melville, NY). The area covered by cells was quantified at each time point using the Wimasis image analysis platform at https://mywim.wimasis.com/index.php?page=Launch&select=Wound_Healing&gr=ibidi (Munich, Germany).

### Matrigel invasion assay

Cell invasion was assayed using the QCM ECMatrix Cell invasion assay (Millipore) using polycarbonate membranes with 8-μm pores and a layer of reconstituted basement membrane matrix. Cells (3 × 10^5^) were suspended in 300 μL serum-free medium and carefully transferred to the upper chambers of the devices. The lower chambers were filled with 500 μL medium containing 10% FBS as an attractant, and the chamber was incubated at 37°C in a humidified atmosphere of 5% CO_2_ for 48 h. Then, non-invading cells were removed from the upper chamber with a cotton swab. Cells that had invaded the polycarbonate membrane to the lower surface were fixed, stained with crystal violet, and quantified by counting five random microscope fields at a magnification of 400×.

### Immunofluorescence microscopy

HT29 cells were seeded on coverslips at 30–40% confluency. After 24 h, the cells were processed for immunofluorescence as described [15] and examined using a laser-scanning confocal microscope (TCS SP2-MP System, Leica, IL) at 1,000× magnification. NP1 was diluted 1:100 to a final concentration of 20 μg/mL, and FITC-conjugated secondary antibody (Jackson ImmunoResearch, West Grove, PA) was diluted 1:500. Cell nuclei were counterstained with DAPI.

### Tumor xenografts

Athymic Balb/c nude mice (NU/NU mice, females, 5 weeks old) were purchased from BioLASCO Taiwan (Yi-Lan, Taiwan). HT29 cells (2 × 10^6^ in 200 μL PBS) were injected subcutaneously into the left side of the mice, and tumor growth was monitored. Tumor volume (V) was estimated from the length (*l*) and width (*w*) of the tumor using the following formula: V = *l* × *w*^*2*^/2.

Treatments were initiated on day 7 after tumor cell inoculation (i.e., when tumor nodules were palpable). Mice were randomized into three groups with six mice per group and injected subcutaneously near the tumor nodules with NP1 (20 μg/dose), isotype-control IgG (20 μg/dose), or PBS one dose every 2 days in a volume of 200 μL for a total of 3 doses (i.e. a 6-day treatment).The NP1 and isotype control IgG used in this study were prepared in the same way. Mice were monitored until day 30 after inoculation, at which time they were sacrificed. Tumors were dissected and weighed. All animal experiments were performed using protocols approved by the Institutional Animal Care and Use Committee of Chang Gung University (CGU10-118). The Committee confirmed that the proposed animal experiments followed the guidelines in the Guide for Laboratory Animal Facilities and Care as promulgated by the Council of Agriculture, Executive Yuan, ROC.

### Cell cycle analysis

HT29 Cells (3 × 10^5^) were plated in 6-well plates and synchronized by starvation for 24 h. Then cells were incubated with medium supplemented with 10% FBS and treated with isotype-control IgG (20 μg/mL) or NP1 (20 μg/mL), respectively. After 6, 12, and 24 h incubation, cells were collected and fixed in 70% ethanol and stored overnight at −20°C. After washing twice with cold PBS, cells were treated with PI/Triton X-100 solution (0.2 mg/mL RNase, 0.1% Triton X-100 and 20 μg/mL propidium iodide (all purchased from Sigma, St. Louis, MO)) at 37°C for 1 h. Cell suspensions were filtered through a 60-μm mesh filter (Spectrum Medical Industries, Laguna Hills, CA). DNA content was measuredwith a flow cytometry (Coulter EPICS XL-MCL, Beckman Coulter) and analyzed by the EXPO32 ADC Software (Beckman Coulter). Data from 10,000 cells were collected for each data file. The cell cycle profile was expressed as the percentage of cells in each cell cycle stage. All experiments were performed at least three times.

### Immunofluorescence staining of peripheral blood mononuclear cells (PBMCs) and red blood cells (RBCs)

PBMCs were harvested and washed three times with PBS. Cells were blocked with 2% BSA in PBS for 1 h at 4°C. The blocking reagent was removed, and NP1 (5 or 20 μg/mL) or HLA Class I antibody (W6/32) in PBS containing 5% BSA was added for 1 h. Unbound antibody was removed, and the cells were washed three times in PBS. Secondary Alexa Fluor® 488–conjugated goat anti-mouse IgG (1:50) was added and incubated for 1 h. Cells were washed three times in PBS, and 10,000 cells were counted using flow cytometry (EPICS XL-MCL, Beckman Coulter).

For human RBC binding assays, human blood was diluted 1:500 in PBS. The diluted RBCs were incubated with NP1 (5 or 20 μg/mL) or mouse anti-human CD47 for 30 min at room temperature. The subsequent procedures were performed as described for PBMCs.

### Statistical analyses

Statistical analyses were carried out using GraphPad Prism 5.0 or SPSS/Windows 12.0 statistical package. Descriptive statistics are expressed as the mean ± standard deviation (SD). Differences in protein expression between groups were evaluated with the nonparametric Mann–Whitney test. Statistical comparisons were performed with *t*-tests for means of independent samples. Categorical variables were expressed as a percentage and analyzed with Pearson’s *chi*-squared test. A *p* value of <0.05 was considered significant.

## Results

### Elevated expression of PLSCR1 in CRC cell lines and various human cancer cells

**Figure 1:**
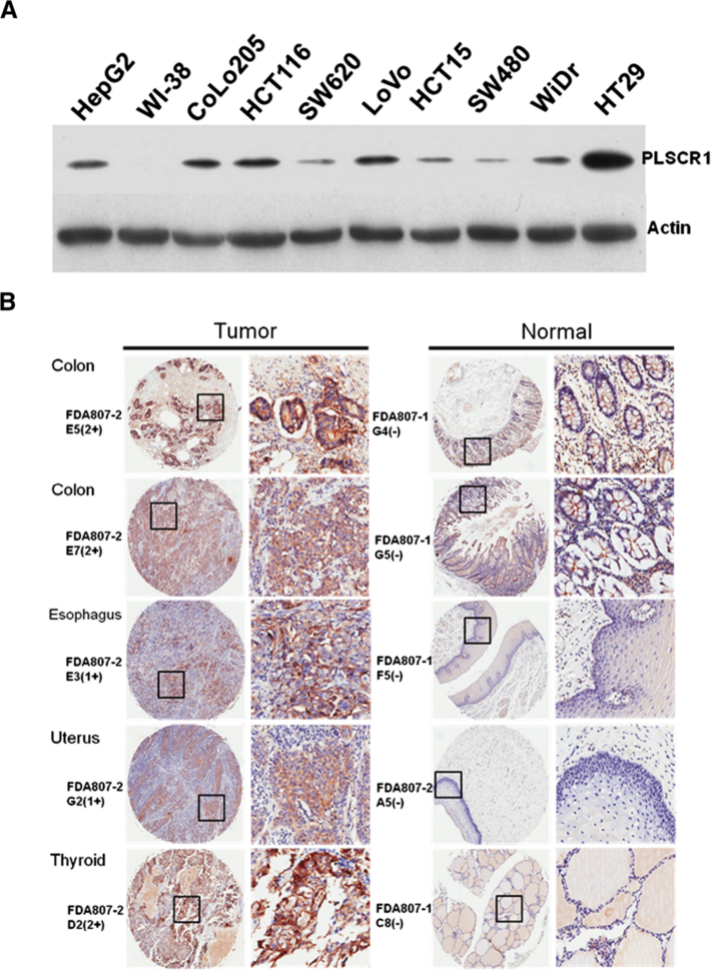
**Elevated expression of PLSCR1 in CRC cell lines and various human cancer cells.** (**A**) Western blot analysis of lysates from ten different cell lines including eight CRC cell lines (CoLo205, HCT116, SW620, LoVo, HCT15, SW480, WiDr, HT29), HepG2 cells (positive control), and one noncancer cell line (WI-38 fibroblasts). The top and bottom blots show PLSCR1 and actin (loading control). (**B**) Tissue microarray analysis of PLSCR1 in multiple normal and tumor tissues. Selected images of colon, esophagus, uterus, and thyroid tissues in tissue arrays representing immunohistochemical staining of PLSCR1 are shown. The boxed areas indicated in the left panels (original magnification, ×10) are enlarged and shown in the right panels (original magnification, ×50).

Western blotting with NP1 was performed on lysates from various cell lines including eight CRC lines (CoLo205, HCT116, SW620, LoVo, HCT15, SW480, WiDr, HT29), HepG2 cells (positive control), and one non-cancer line (WI-38 fibroblasts). PLSCR1 was expressed in all CRC cell lines examined and was most strongly expressed in HT29, moderately expressed in HCT116, CoLo205, LoVo, and WiDr, and expressed at very low levels in SW620 and SW480 (Figure 1A). PLSCR1 was undetectable in WI-38 fibroblasts.

To investigate PLSCR1 expression in different solid cancers and adjacent normal tissues, we analyzed immunohistochemistry tissue arrays with NP1 using 54 tumor cases from 27 types of human organs, plus 90 cases from 24 common types of normal human organs. PLSCR1 was highly expressed in colon adenocarcinoma, medullary thyroid carcinoma, and transitional bladder carcinomas. Tissues such as pancreatic adenocarcinoma, esophageal adenocarcinoma, rectal adenocarcinoma, and squamous cell carcinoma of the cervix showed low PLSCR1 expression (Additional file 1: Table S1 & Additional file 2: Figure S1). Only two (adrenal gland and liver) of the 90 normal tissues cases showed low expression of PLSCR1. Figure 1B shows representative examples of PLSCR1 expression in tissue arrays for colon, esophagus, uterus, and thyroid tissues.

We showed that NP1 bound to the cell membrane of HT 29 cells, in contrast to isotype-control IgG, which did not bind to cells with immunofluorescence analysis (Additional file 3: Figure S2). We next examined PLSCR1 expression in human PBMCs and RBCs with flow cytometry. NP1 did not significantly bind to PBMCs compared with anti-HLA Class I, a positive control (panel A). NP1 also did not significantly bind to RBCs compared with anti-human CD47 (panel B). Thus, NP1 did not bind to or otherwise affect PBMCs and RBCs when administered intravenously at a concentration of 5–20 μg/mL (Additional file 4: Figure S3).

### Treatment with NP1 reduces tumorigenic properties of CRC cells *in vitro* and *in vivo*

**Figure 2:**
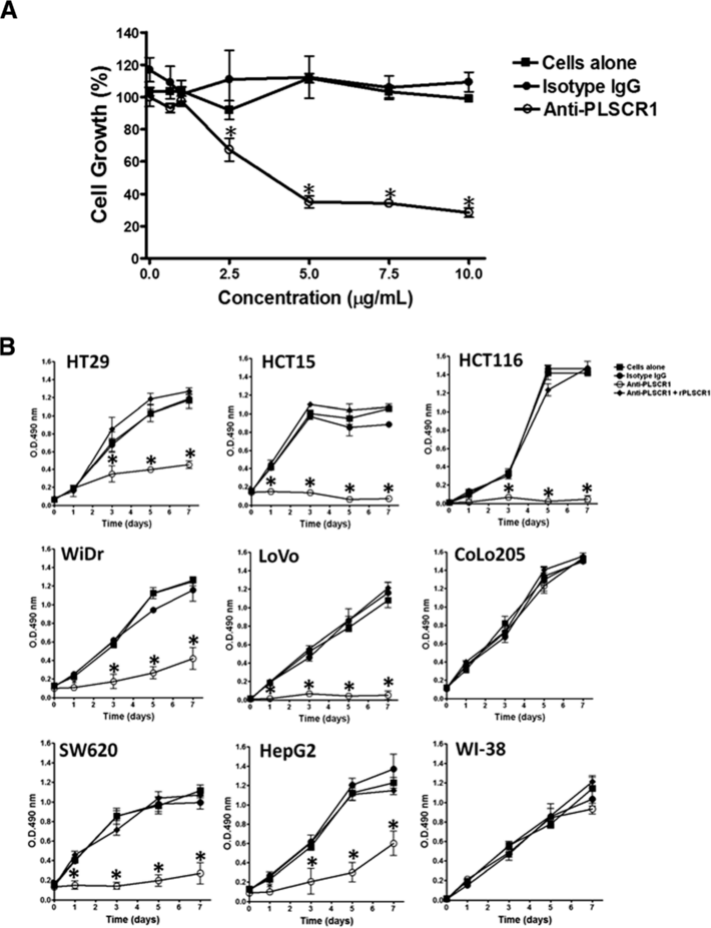
**Anti-PLSCR1 represses the proliferation of CRC cells *in vitro*.** (**A**) The proliferation of HT29 cells was inhibited by treatment with NP1 in a dose-dependent manner. Error bars show the SD. * *p* < 0.05. (**B**) Growth curves of CRC cells (HT29, HCT15, HCT116, WiDr, LoVo, CoLo205, SW620, HepG2 and WI-38) treated with isotope-control IgG, NP1, or NP1 previously neutralized with recombinant PLSCR1 are shown.

To inhibit PLSCR1 function, we used NP1 to treat CRC cell lines, and proliferation was compared with that of cells treated with isotype-control IgG. NP1 attenuated growth of HT29 cells in a dose-dependent manner (Figure 2A). Figure 2B shows that treatment with NP1 (5 μg/mL) attenuated growth of HT29, WiDr, HCT15, LoVo, SW620, HepG2, and HCT116 cells, whereas it did not affect the growth of CoLo205 cells. To rule out the possibility of a non-specific reaction, we treated the cells with exogenous recombinant PLSCR1 (Additional file 5: Figure S4; Additional file 6: Method S1) and NP1, and the proliferative capacity was restored (Figure 2B).

**Figure 3:**
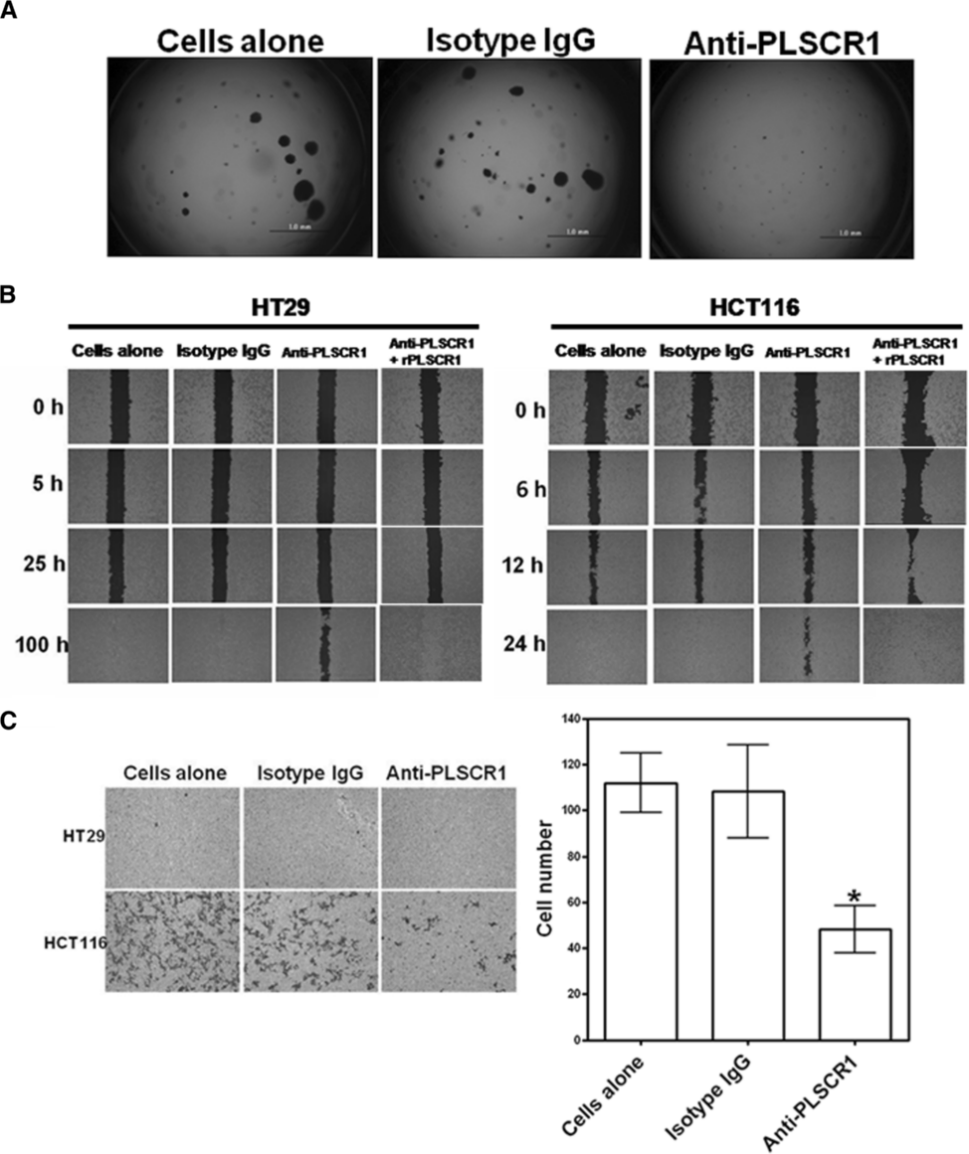
**Effects of anti-PLSCR1 on anchorage-independent colony formation, migration, and invasion of CRC cells.** (**A**) The anchorage-independent colony-forming ability of HT29 cells treated with isotype-control IgG or NP1 was compared using the soft-agar cell transformation assay. Scale bar = 1.0 mm. (**B**) Migration of HT29 and HCT116 cells treated with NP1 is impaired compared to cells treated with isotype-control IgG. Cell-covered areas were quantified using the Wimasis image analysis platform. (**C**) Invasion assays with HT29 and HCT116 cells. The chamber was incubated at 37°C for 48 hours and cells that had invaded the polycarbonate membrane to the lower surface were quantified by counting five random microscope fields. Representative photomicrographs of filters (left panel) and quantitative analysis of the assay with HCT116 cells (right panel) are shown. Each bar represents the mean ± SD calculated from three independent experiments.

Next we assessed the ability of CRC cells to form anchorage-independent colonies. Colonies formed by untreated HT29 cells were much larger than those formed by NP1-treated cells, suggesting a substantial potential for NP1 to inhibit the ability of HT29 colony formation (Figure 3A). We also examined the effects of anti-PLSCR1 on two important tumorigenic characteristics of CRC cells—migration and invasion. HCT116 cell migration and invasion were severely impaired by NP1 compared to the same cells treated with PBS or isotype-control IgG. Migration decreased by 20% at 24 h (Figure 3B), and invasion decreased by ~56% at 48 h (Figure 3C). On the other hand, migration of HT29 cells decreased only after incubating with NP1 for 100 h (Figure 3B). In addition, HT29 cells did not invade the polycarbonate membrane to the lower surface after 48 h of incubation (Figure 3C). It is possible that HT29 cells, which are derived from Dukes’ B cells, are incapable of invasion. To rule out the possibility of a non-specific reaction of migration, we treated the cells with exogenous recombinant PLSCR1 and NP1, and the migration capacity was restored (Figure 3B).

**Figure 4:**
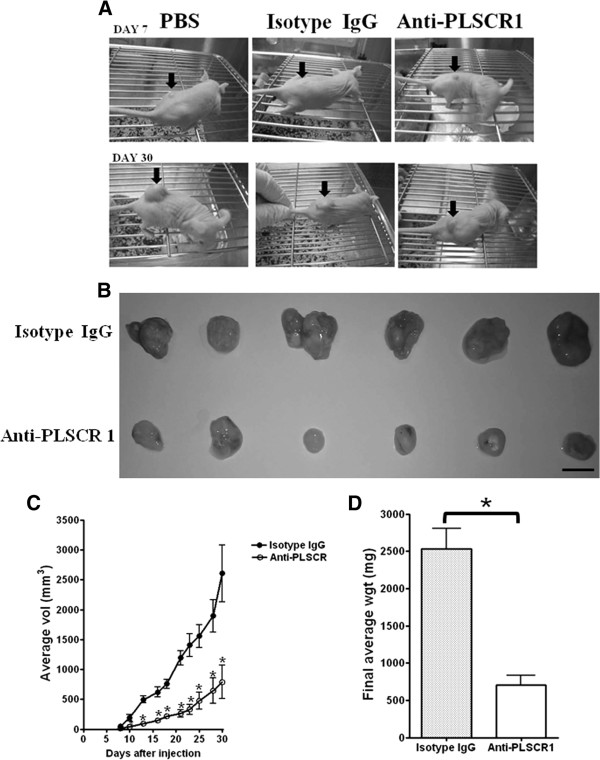
**Anti-PLSCR1 inhibits growth of xenogenic transplanted HT29 tumors.** (**A**) Representative photographs of a mouse from each group at day 7 and day 30 are shown. Arrows indicate tumor nodules. (**B**) Tumors dissected from groups treated with isotype-control IgG or NP1 are shown below. (**C**) Tumor growth curve were evaluated until day 30 and (**D**) final average tumor weight from mice treated with isotype-control IgG or NP1. Data are mean ± SD of 12 samples from twice experiments. * *p* < 0.05.

We next investigated if NP1 inhibits growth of xenogenic transplanted HT29 tumors. We examined the ability of NP1 to suppress tumor growth in NU/NU mice. Six mice were inoculated with HT29 cells and then treated with non-specific isotype-control IgG every other day as a control group, and another six mice were inoculated with HT29 cells and then injected with NP1 every other day as an experimental group. The experiments were evaluated twice. The group treated with NP1 showed suppressed tumor growth. A representative photograph of a mouse from each group is shown in Figure 4A. At 30 days post-inoculation, the tumors were excised and weighed. Mice treated with NP1 showed a dramatic decrease in average tumor size at day 10 after inoculation with HT29 cells compared with untreated mice (Figure 4B). Tumors in mice treated with isotype-control IgG grew an average of 3.3-fold larger than tumors in mice treated with NP1. At 23 days after NP1 treatment, average tumor size was 791 ± 689 mm^3^, whereas that of mice treated with isotype-control IgG was 2607 ± 1164 mm^3^ (Figure 4C). The average tumor weight in mice treated with NP1 was 703 ± 330 mg, whereas that in mice treated with isotype-control IgG was 2532 ± 685 mg, a 3.6-fold difference (Figure 4D). The tumors in HT29-injected mice first appeared at the injection site at day 7, and the final average tumor weights and tumor growth curves indicated that NP1 suppressed the growth of HT29-derived tumors *in vivo*.

### NP1 induces G1/S arrest in the CRC cell cycle

**Figure 5:**
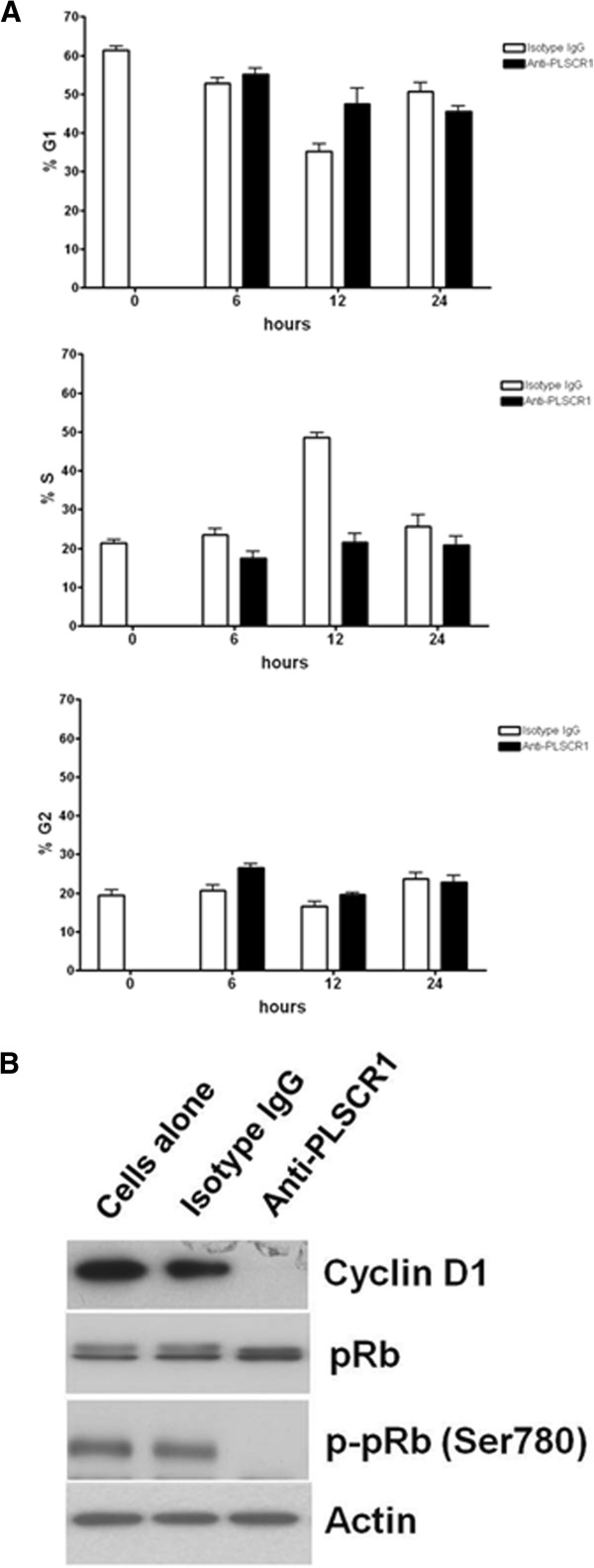
**Anti-PLSCR1 induces G1/S arrest and activates the tumor suppressor protein, pRb.** (**A**) Cell-cycle distributions of HT29 cells at different time points after the addition of NP1 are shown. DNA content was analyzed by the EXPO32 ADC Software (EPICS XL-MCL, Beckman Coulter). Data are presented as the mean ± SD calculated from three experiments. Cells treated with NP1 accumulated higher percentage of cells in G1 at 12 h compared with cells treat with isotype-control IgG. (**B**) Western blot analysis of cyclin D1, total pRb, and phosphorylated pRb after cells were treated with NP1 or isotype-control IgG is shown. Actin was used as a loading control.

To study the mechanism by which NP1 reduces cell growth and viability, HT29 cells were synchronized with serum starvation for 24 h and then treated with NP1. At the time NP1 was added (time = 0 h), the cell cycle distribution of HT29 cells was as follows: 61% in G_0_/G_1_, 21% in S, and 18% in G2/M (Figure 5A). In cells treated with isotype-control IgG, the fraction of cells in the G1 phase decreased, and the fraction in the S phase increased at 6 and 12 h, indicating cell cycle progression from G1 to S. At 24 h, the S population decreased and the G1 population increased, reflecting cell cycle progression from S to G1. On the other hand, addition of NP1 resulted in a higher percentage of cells that accumulated in G1 at 12 h (Figure 5A) compared with cells treated with isotype-control IgG. For cells treated with isotype-control IgG at 6 and 12 h, the relatively higher numbers of cells in S correlated with lower numbers in G1, suggesting that NP1 inhibited the progression from G1 to S, resulting in G1/S arrest.

Cyclin D1 plays a major role in cell cycle regulation, and its overexpression shortens the time spent in G1; thus, decreasing the amount of cyclin D1 may block progression to S phase. Therefore, we investigated the level of cyclin D1 in NP1 treated cells and found that it was undetectable (Figure 5B). Cyclin D1 expression was similar in untreated and isotype control IgG treated cells.

In proliferating cells, accumulation of the cyclin D1 dependent kinase complex is important for deactivating the tumor suppressor protein, retinoblastoma (pRb), via hyperphosphorylation. Therefore, low expression of cyclin D1 may result in hypophosphorylated pRb, the active form of this tumor suppressor. The level of pRb was similar in NP1 treated, isotype-control IgG treated, and untreated cells, but Ser^780^ in pRb was dephosphorylated in NP1 treated cells (Figure 5B).

### Elevated expression of PLSCR1, Shc, Src, and cyclin D1 in CRC tissues compared with adjacent normal tissues

**Figure 6:**
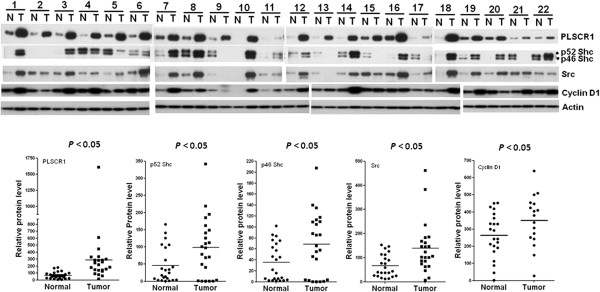
**Western blot analysis of PLSCR1, Shc, Src, and cyclin D1 expression in colorectal tissue pairs.** Twenty-two tissue pairs were utilized to evaluate protein expression. The expression of each protein in tumor (T) *versus* normal (N) tissues was evaluated with the nonparametric Mann–Whitney test (*p* < 0.05). The raw data points are presented as a scatter plot, and each mean value is indicated with a horizontal line.

Multiple proline-rich motifs (PXXP and PPXY) in the N-terminal domain of PLSCR1 suggest that this protein may interact with other proteins with Src homology 3 (SH3) and WW domains [16]. A possible role for PLSCR1 in signaling through growth factor receptors was suggested by an increase in kinase activity of cellular c-Src through EGFR [12]. We hypothesized that PLSCR1 may functionally interact with other molecules containing SH3 domains or with EGFR-related molecules. Thus, we collected tissue pairs of adjacent normal and tumor tissues that overexpressed PLSCR1 and examined the expression of the adaptor protein Shc, Src, and cyclin D1. Twenty-two pairs of colorectal tissues from 22 different CRC patients were examined with western blotting. In most cases, PLSCR1, Shc, Src, and cyclin D1 were expressed at higher levels in CRC tissues than in the corresponding adjacent normal tissues (*p* < 0.05; Figure 6).

### Anti-PLSCR1 represses tumorigenesis by inhibiting EGFR-related downstream signaling

**Figure 7:**
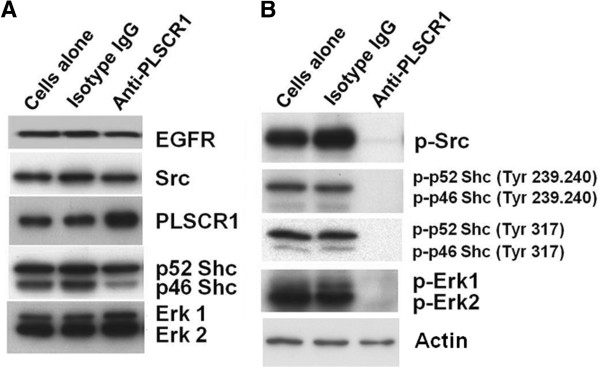
**Anti-PLSCR1 represses tumorigenesis by inhibiting EGFR-related downstream signaling.** (**A**) The expression of EGFR, Src, PLSCR1, Shc, and Erks was evaluated with western blotting after the cells were treated with NP1 or isotype-control IgG. (**B**) Phosphorylation of Src, Shc, and Erks decreased following NP1 treatment in HT29 cells.

We further extended our evaluation of EGFR-related downstream effectors in HT29 cells by examining expression of EGFR, PLSCR1, Shc, Src, Erk1/2, and the levels of phosphorylation of these proteins in response to NP1. NP1 repressed the expression of cyclin D1 and activated pRb via dephosphorylation as shown in Figure 5B. Figure 7A shows that the expression of EGFR, Src, Shc, and Erk in HT29 cells was not affected by NP1 treatment. However, NP1 decreased the phosphorylation of Src, Shc, and Erk (Figure 7B). In addition, NP1 decreased the phosphorylation of Shc on three different tyrosine residues, namely Tyr^239^, Tyr^240^, and Tyr ^317^, which are involved in interactions between Shc and other proteins.

## Discussion

CRC is the fourth leading cause of mortality from cancer [17]. Increasing efforts over the past 20 years have focused on developing more chemotherapeutic agents and monoclonal antibodies for this disease, and the median overall survival rate has increased. More recent studies have emphasized not only the efficacy of combining traditional chemotherapeutic agents but also how best to develop effective targets for therapy. Monoclonal antibodies against vascular endothelial growth factor (VEGF) and EGFR have significantly improved the overall survival of CRC patients [18]. New targets must be developed because many patients are not candidates for anti-VEGF or anti-EGFR therapy owing to *KRAS* mutations [19]. In the present study, we provide evidence that anti-PLSCR1 represses the tumorigenic potential of CRC cells. PLSCR1 plays a major role both in movement of membrane phospholipids and in cell signaling. PLSCR1 is highly expressed in CRC tissues and at low levels in adjacent normal tissues [2]. Thus, PLSCR1 overexpression may promote cell proliferation and transformation, and blocking PLSCR1 function in transformed cells may reduce their tumorigenic properties. PLSCR1 activity affects EGFR-related signaling and its downstream effectors, including the adaptor protein Shc, Src, and Erk kinases in fibroblasts and epithelial cells [12]. In intestinal epithelial cells, EGF induces phosphoinositide 3 kinase (PI3K) activity and cyclin D1 expression, suggesting that the PI3K/AKT pathway transduces proliferative signals between growth factor receptors and the cell cycle machinery [20].

Our present study showed that blocking PLSCR1 activity with a corresponding antibody in CRC cells resulted in downregulation of cyclin D1 and reduced phosphorylation of signaling-related molecules, such Shc, Src, and Erk kinases (Figures 5 and 7). These results are consistent with results from previous studies that strongly indicate that PLSCR1 is oncogenic during EGFR-related signaling and firmly support the idea that activation of the EGFR pathway alone is sufficient for transformation. Furthermore, we found that anti-PLSCR1 suppressed cyclin D1 expression and subsequently produced hypophosphorylated pRb, and this resulted in arrest at the G1/S boundary of the cell cycle (Figure 5). Therefore, we propose that tumorigenesis caused by PLSCR1 involves EGFR-related signaling. Consistent with this idea, anti-PLSCR1 suppressed EGFR-related signaling, thereby inhibiting transformation.Our results also suggest that PLSCR1 is important for CRC cell proliferation, migration, and invasion (Figures 2 &3). Similarly, it was recently reported that silencing of PLSCR1 by siRNA could inhibit the proliferation, adhesion, migration and invasion of Lovo cells [21]. However, the mechanism by which PLSCR1 promotes tumorigenesis remains unclear. We suggest that the tumorigenic properties of CRC cells are due to altered EGFR function. PLSCR1 has been reported to be regulated by EGFR [12], and upon EGF stimulation PLSCR1 is phosphorylated by c-Src [12]. In addition, immunoprecipitation and western blotting have been used to show a transient increase in the physical association of PLSCR1 with both EGFR and Shc [22]. The EGFR-related pathway was also identified as a transformation-related pathway in multiple cancer types and appears to promote growth of solid tumors [23].

PLSCR1 activity was suppressed by anti-PLSCR1 *in vitro* and *in vivo*. Whether PLSCR1 activity can be suppressed by other antibodies against PLSCR1 (or more interestingly by other non-antibody inhibitors) is an important issue. In a recent study, Bateman and collaborators performed extensive similarity searches to develop a molecular model of the PLSCR family and showed that most members possess an N-terminal stretch of 100–250 natively unfolded residues that may function as activation domains or protein-protein interaction domains [24]. To gain new insight into other potential functional domains of PLSCR1, to clearly understand PLSCR1 function, and to develop PLSCR1 inhibitors, a predicted molecular structure is required in concert with site-directed mutagenesis studies.

In this study, we found that CoLo205 CRC cells expressed high levels of PLSCR1, but treatment of these cells with NP1 did not affect their growth (Figure 1 and 2B). The CoLo205 cell line is derived from distant metastasis cells of Duke’s D tissue, and these advanced CRC cells may possess an alternate route for survival in the presence of anti-PLSCR1. Studies have shown that the integrity of the EGFR-activated downstream intracellular signal transduction machinery may influence the response to therapeutic agents. In this regard, evidence has suggested that cancer cells may escape growth inhibition by using an alternate growth pathway or by constitutively activating downstream signaling effectors. For example, human A431 squamous cancer cells can acquire resistance to monoclonal antibodies against EGFR by increasing tumor-induced angiogenesis owing to constitutive overexpression of VEGF [25]. In addition, human glioblastoma cells efficiently counter the anti-proliferative effects of EGFR inhibitors by continuous activation of the anti-apoptotic PI3K/AKT signaling pathway *in vitro*[26].

Because many growth-controlling pathways may be altered in cancer cells, a combination of therapeutics targeting two or more pathways should be tested in a clinical setting to develop a multi-target therapy that is based on a rational approach to the alterations that are present in an individual cancer patient. In preclinical experimental models, significant and sustained anti-tumor activity *in vitro* and *in vivo* can be obtained with the combination of anti-EGFR agents and other anti-signaling agents, such as inhibitors of the cAMP-dependent protein kinase [27,28], an antisense oligonucleotide targeting VEGF [29], or the monoclonal antibody trastuzumab, which targets ErbB-2 [30,31]. Estimating the cooperative inhibitory effect of NP1 in combination with other anti-signaling agents on human CRC cell growth will be valuable.

A typical antibody dependent cell-mediated cytotoxicity (ADCC) is usually mediated by the Fc of antigen specific antibody binding to the CD16, an Fc receptor, on natural killer (NK) cells. Once the Fc receptor binds to the Fc region of IgG, the NK cell releases cytokines such as IFN-γ, and cytotoxic granules containing perforin and granzymes that enter the target cell and promote cell death by triggering apoptosis. In this study, NP1 appeared to be the specific antibody which recognizes cancer cells, such might results in ADCC. In contrast, the isotype-control IgG, sharing the same Fc region, failed to induce ADCC and had no effect on tumor growth. Although the xeno-grafted tumor model in nude mice is common to examine anti-tumor treatment due to the avoidance of the T cell mediated xeno-rejection responses, the NK cells in nude mice still exhibit Fcγ receptors. Therefore, NP1 triggering ADCC which involves activation of NK cells could be another potential mechanism. It will be much help of utilizing NK and T-cell depleted hosts to clarify the issue in the future studies.

The exact mechanism of PLSCR1 in tumorigenesis is still controversial. Although we provide evidences to support the ability of PLSCR1for promote tumor development. However, some recent reports have different findings. Kasukabe et al. and Nakamaki et al. showed that expression of the PLSCR1 gene contributes to the differentiation and proliferation of leukemia cells. Yokoyama et al. indicated that mRNA level of PLSCR1 is a prognostic factor for acute myelogenous leukemia [32-34], and Huang et al. further demonstrated that PLSCR1 plays the antagonistic role regarding leukemia development and PLSCR1 induction increased cellular sensitivity to apoptosis [35]. Silverman et al. found that stably over-expressing PLSCR1 in human ovarian cancer cell line did suppress cell growth *in vivo*, whereas it had no impact on cellular growth rate *in vitro*[8]. These studies suggest that alternative biological functions may possess in various types of cancer for this protein.

In summary, *in vitro* and *in vivo* data suggest that anti-PLSCR1 may have therapeutic potential for CRC. The ability of anti-PLSCR1 to suppress proliferation in cell culture and reduce the *in vivo* growth of HT29 cells for a prolonged period suggests that anti-PLSCR1 is likely relatively effective in suppressing CRC. The tumor growth may be repressed via blocking of PLSCR1-related tumorigenic signals and/or antibody dependent cell-mediated cytotoxicity. In addition, NP1 did not bind to PBMCs and RBCs and is thus unlikely to cause side effects when administered intravenously (Additional file 4: Figure S3). A range of EGFR-targeted agents and agents with an intracellular site of action have been developed [36-39]. Determining whether anti-PLSCR1 has a therapeutic value similar to other anti-EGFR drugs, such as gefitinib, will be valuable.

## Abbreviations

PLSCR1: Phospholipid scramblase 1; CRC: Colorectal carcinomas; FBS: Fetal bovine serum; HLA: Human leukocyte antigen; ELISA: Enzyme-linked immunosorbent assay; PBMC: Peripheral blood mononuclear cell; RBC: Red blood cell; pRb: Retinoblastoma; SH3: Src homology 3; VEGF: Vascular endothelial growth factor; PI3K: Phosphoinositide 3 kinase; ADCC: Antibody dependent cell-mediated cytotoxicity; NK: Natural killer cell.

## Competing interests

The authors declare that they have no competing interests as defined by *Journal of Translational Medicine* or other interests that might be perceived to influence the results and discussion reported in this paper.

## Authors’ contributions

FCW participated in the design of the study, data acquisition and analysis, and drafting of the manuscript. CCY performed western blot analysis and cell cycle analysis. CKT carried out the cell proliferation assay and anchorage-independent transformation assay, participated in the design of the study. SCR participated in the design of the mouse model studies, analysis and interpretation of data. KYB participated in immunofluorescence staining and performed the statistical analysis. CYS performed the mouse model studies. CYP participated in immunohistochemical analysis of the tissue array. WWS participated in the cell proliferation assay. CEC conceived of the study, and participated in its design and coordination and helped to draft the manuscript. All authors read and approved the final manuscript.

## Supplementary Material

Additional file 1 **Table S1.** Tissue microarray analysis of PLSCR1 expression in multiple normal and tumor tissues. Click here for file

Additional file 2 **Figure S1.** Immunohistochemical examination for PLSCR1 expression in 24 common types of normal tissues and 27 types of human tumor tissues. Click here for file

Additional file 3 **Figure S2.** Immunofluorescence staining of HT29 cells with Anti-PLSCR1 antibody. Click here for file

Additional file 4 **Figure S3.** Binding assays of anti-PLSCR1 to human peripheral blood mononuclear cells (PBMCs) and red blood cells (RBCs). Click here for file

Additional file 5 **Figure S4.** Development of recombinant PLSCR1 and identification of PLSCR1 by mass spectrometry. Click here for file

Additional file 6 Preparation of recombinant PLSCR1 protein. Click here for file
