# Supplementary material for: Blockade of phospholipid scramblase 1 with its N-terminal domain antibody reduces tumorigenesis of colorectal carcinomas in vitro and in vivo
Source: J Transl Med. 2012 Dec 24;10:254. doi: 10.1186/1479-5876-10-254 (PMC3551821; doi:10.1186/1479-5876-10-254)
Supplement: Additional file 1 — Table S1. Tissue microarray analysis of PLSCR1 expression in multiple normal and tumor tissues. [file 1479-5876-10-254-S1.doc]

**Supplemental table 1. Tissue microarray analysis of PLSCR1 expression in multiple normal and tumor tissues.**

**FDA807-1**

| Pos | No. | Gender | Age | Organ | Pathology diagnosis | Type | PLSCR1 expression |
| --- | --- | --- | --- | --- | --- | --- | --- |
| A1 | 1 | F | 2 | Cerebrum | Normal cerebrum tissue | Normal | **−** |
| A2 | 2 | F | 50 | Cerebrum | Normal cerebrum tissue | Normal | **−** |
| A3 | 3 | F | 42 | Cerebrum | Normal cerebrum tissue | Normal | **−** |
| A4 | 4 | F | 24 | Cerebellum | Normal cerebellum tissue | Normal | **−** |
| A5 | 5 | F | 58 | Cerebellum | Normal cerebellum tissue | Normal | **−** |
| A6 | 6 | F | 8 | Cerebellum | Normal cerebellum tissue | Normal | **−** |
| A7 | 7 | F | 14 | Adrenal gland | Normal adrenal gland tissue | Normal | **−** |
| A8 | 8 | M | 1 Mon. | Adrenal gland | Normal adrenal gland tissue | Normal | **−** |
| A9 | 9 | F | 21 | Adrenal gland | Normal adrenal gland tissue | Normal | + |
| B1 | 10 | F | 41 | Ovary | Normal ovary tissue | Normal | **−** |
| B2 | 11 | F | 49 | Ovary | Normal ovary tissue | Normal | **−** |
| B3 | 12 | F | 49 | Ovary | Normal ovary tissue | Normal | **−** |
| B4 | 13 | F | 35 | Pancreas | Normal pancreas tissue | Normal | **−** |
| B5 | 14 | F | 21 | Pancreas | Normal pancreas tissue | Normal | **−** |
| B6 | 15 | M | 35 | Pancreas | Normal pancreas tissue | Normal | **−** |
| B7 | 16 | F | 50 | Parathyroid gland | Normal thyroid gland tissue | Normal | **−** |
| B8 | 17 | F | 40 | Parathyroid gland | Normal thyroid gland tissue | Normal | **−** |
| B9 | 18 | M | 50 | Parathyroid gland | Normal parathyroid gland tissue | Normal | **−** |
| C1 | 19 | F | 40 | Pituitary gland | Normal hypophysis tissue | Normal | **−** |
| C2 | 20 | M | 17 | Pituitary gland | Normal hypophysis tissue | Normal | **−** |
| C3 | 21 | F | 15 | Pituitary gland | Normal hypophysis tissue | Normal | **−** |
| C4 | 22 | M | 30 | Testis | Normal testis tissue | Normal | **−** |
| C5 | 23 | M | 74 | Testis | Normal testis tissue | Normal | **−** |
| C6 | 24 | M | 65 | Testis | Normal testis tissue | Normal | **−** |
| C7 | 25 | M | 37 | Thyroid gland | Normal thyroid gland tissue | Normal | **−** |
| C8 | 26 | M | 22 | Thyroid gland | Normal thyroid gland tissue | Normal | **−** |
| C9 | 27 | M | 50 | Thyroid gland | Normal thyroid gland tissue | Normal | **−** |
| D1 | 28 | F | 35 | Breast | Normal breast tissue | Normal | **−** |
| D2 | 29 | F | 33 | Breast | Normal breast tissue | Normal | **−** |
| D3 | 30 | F | 40 | Breast | Normal breast tissue | Normal | **−** |
| D4 | 31 | F | 2 | Spleen | Normal spleen tissue | Normal | **−** |
| D5 | 32 | M | 35 | Spleen | Normal spleen tissue | Normal | **−** |
| D6 | 33 | M | 30 | Spleen | Normal spleen tissue | Normal | Loss |
| D7 | 34 | F | 15 | Tonsil | Normal tonsil tissue | Normal | **−** |
| D8 | 35 | F | 18 | Tonsil | Normal tonsil tissue | Normal | **−** |
| D9 | 36 | M | 50 | Tonsil | Normal tonsil tissue | Normal | **−** |
| E1 | 37 | M | 7 Mon. | Thymus gland | Normal thymus gland tissue | Normal | **−** |
| E2 | 38 | F | 15 | Thymus gland | Normal thymus gland tissue | Normal | **−** |
| E3 | 39 | M | 16 | Thymus gland | Normal thymus gland tissue | Normal | **−** |
| E4 | 40 | M | 56 | Bone marrow | Normal myeloid tissue | Normal | **−** |
| E5 | 41 | F | 61 | Bone marrow | Normal myeloid tissue | Normal | **−** |
| E6 | 42 | M | 70 | Bone marrow | Normal myeloid tissue | Normal | **−** |
| E7 | 43 | M | 24 | Lung | Normal lung tissue | Normal | **−** |
| E8 | 44 | M | 42 | Lung | Normal lung tissue | Normal | **−** |
| E9 | 45 | M | 48 | Lung | Normal lung tissue | Normal | **−** |
| F1 | 46 | M | 56 | Heart | Normal cardiac muscle tissue | Normal | **−** |
| F2 | 47 | F | 42 | Heart | Normal cardiac muscle tissue | Normal | **−** |
| F3 | 48 | F | 35 | Heart | Normal cardiac muscle tissue | Normal | **−** |
| F4 | 49 | M | 35 | Esophagus | Normal esophagus tissue | Normal | **−** |
| F5 | 50 | M | 24 | Esophagus | Normal esophagus tissue | Normal | **−** |
| F6 | 51 | F | 42 | Esophagus | Normal esophagus tissue | Normal | **−** |
| F7 | 52 | M | 48 | Stomach | Normal stomach tissue | Normal | **−** |
| F8 | 53 | M | 35 | Stomach | Normal stomach tissue | Normal | **−** |
| F9 | 54 | M | 56 | Stomach | Normal stomach tissue | Normal | **−** |
| G1 | 55 | M | 40 | Small intestine | Normal small intestine tissue | Normal | **−** |
| G2 | 56 | M | 25 | Small intestine | Normal small intestine tissue | Normal | **−** |
| G3 | 57 | M | 35 | Small intestine | Normal small intestine tissue | Normal | **−** |
| G4 | 58 | M | 62 | Colon | Normal colon tissue | Normal | **−** |
| G5 | 59 | M | 30 | Colon | Normal colon tissue | Normal | **−** |
| G6 | 60 | M | 35 | Colon | Normal colon tissue (smooth muscle tissue) | Normal | **−** |
| G7 | 61 | M | 40 | Liver | Normal liver tissue | Normal | **−** |
| G8 | 62 | F | 35 | Liver | Normal liver tissue | Normal | **−** |
| G9 | 63 | M | 35 | Liver | Normal liver tissue | Normal | + |
| H1 | 64 | M | 50 | Salivary gland | Normal salivary gland tissue | Normal | **−** |
| H2 | 65 | M | 77 | Salivary gland | Normal salivary gland tissue | Normal | **−** |
| H3 | 66 | F | 34 | Salivary gland | Normal salivary gland tissue | Normal | **−** |
| H4 | 67 | F | 14 | Kidney | Normal kidney tissue | Normal | **−** |
| H5 | 68 | M | 48 | Kidney | Normal kidney tissue | Normal | **−** |
| H6 | 69 | F | 50 | Kidney | Normal kidney tissue | Normal | **−** |
| H7 | 70 | M | 27 | Prostate | Normal prostate tissue | Normal | **−** |
| H8 | 71 | M | 43 | Prostate | Normal prostate tissue | Normal | **−** |
| H9 | 72 | M | 28 | Prostate | Normal prostate tissue | Normal | **−** |
| – | – | M | 58 | Skin | Malignant melanoma (tissue marker) | Malignant | **−** |

FDA807-2

| Pos | No. | Gender | Age | Organ | Pathology  diagnosis | Grade^1^ | Stage | TNM | Type | PLSCR1 expression |
| --- | --- | --- | --- | --- | --- | --- | --- | --- | --- | --- |
| A1 | 1 | F | 21 | Uterus | Normal endometrium tissue | – | – | – | Normal | **−** |
| A2 | 2 | F | 21 | Uterus | Normal endometrium tissue | – | – | – | Normal | **−** |
| A3 | 3 | F | 18 | Uterus | Normal endometrium tissue | – | – | – | Normal | **−** |
| A4 | 4 | F | 30 | Uterus | Cancer adjacent normal cervix tissue | – | – | – | NAT^2^ | **−** |
| A5 | 5 | F | 50 | Uterus | Cancer adjacent normal cervix tissue | – | – | – | NAT | **−** |
| A6 | 6 | F | 31 | Uterus | Cancer adjacent normal cervix tissue with focal hyperplasia of squamous epithelium | – | – | – | NAT | **−** |
| A7 | 7 | F | 21 | Striated muscle | Normal skeletal muscle tissue | – | – | – | Normal | **−** |
| A8 | 8 | M | 30 | Striated muscle | Normal skeletal muscle tissue | – | – | – | Normal | **−** |
| A9 | 9 | M | 35 | Striated muscle | Normal skeletal muscle tissue | – | – | – | Normal | **−** |
| B1 | 10 | F | 18 | Skin | Normal skin tissue | – | – | – | Normal | **−** |
| B2 | 11 | M | 37 | Skin | Normal skin tissue | – | – | – | Normal | **−** |
| B3 | 12 | M | 34 | Skin | Normal skin tissue | – | – | – | Normal | **−** |
| B4 | 13 | F | 15 | Nerve | Normal nervous tissue (sparse) | – | – | – | Normal | **−** |
| B5 | 14 | F | 27 | Nerve | Normal nervous tissue | – | – | – | Normal | **−** |
| B6 | 15 | M | 31 | Nerve | Normal nervous tissue | – | – | – | Normal | **−** |
| B7 | 16 | M | 48 | Lung | Normal mesothelium tissue (lung tissue sparse) | – | – | – | Normal | **−** |
| B8 | 17 | M | 22 | Lung | Normal mesothelium and lung tissue | – | – | – | Normal | **−** |
| B9 | 18 | M | 47 | Lung | Normal mesothelium tissue (lung tissue) | – | – | – | Normal | **−** |
| C1 | 19 | F | 59 | Cerebrum | Glioblastoma | – | – | – | Malignant | **−** |
| C2 | 20 | F | 65 | Cerebrum | Atypical meningioma | – | – | – | Malignant | **−** |
| C3 | 21 | F | 15 | Cerebrum | Malignant ependymoma | – | – | – | Malignant | **−** |
| C4 | 22 | M | 55 | Cerebrum | Malignant oligodendroglioma | – | – | – | Malignant | **−** |
| C5 | 23 | F | 49 | Ovary | Serous papillary adenocarcinoma | 3 | II | T3N0M0 | Malignant | **−** |
| C6 | 24 | F | 54 | Ovary | Mucinous papillary adenocarcinoma | 2 | IIa | T2aN0M0 | Malignant | **−** |
| C7 | 25 | M | 25 | Pancreas | Islet cell carcinoma | – | II | T2N0M0 | Malignant | **−** |
| C8 | 26 | M | 64 | Pancreas | Adenocarcinoma | 3 | II | T3N0M0 | Malignant | + |
| C9 | 27 | M | 32 | Testis | Seminoma | – | – | – | Malignant | **−** |
| D1 | 28 | M | 25 | Testis | Embryonal carcinoma | – | II | T4N1M0 | Malignant | **−** |
| D2 | 29 | F | 33 | Thyroid | Medullary carcinoma | – | II | T3N0M0 | Malignant | 2+ |
| D3 | 30 | F | 36 | Thyroid gland | Papillary carcinoma | – | III | T2aN1M0 | Malignant | **−** |
| D4 | 31 | F | 28 | Breast | Intraductal carcinoma | – | IIb | T3N0M0 | Malignant | **−** |
| D5 | 32 | F | 43 | Breast | Intraductal carcinoma with early infiltrate | – | IIa | T2N0M0 | Malignant | **−** |
| D6 | 33 | F | 39 | Breast | Invasive ductal carcinoma | 2 | IIb | T2N1M0 | Malignant | **−** |
| D7 | 34 | M | 27 | Spleen | Diffuse large B-cell lymphoma | – | – | – | Malignant | **−** |
| D8 | 35 | M | 44 | Lung | Small cell undifferentiated carcinoma | – | II | T2N1M0 | Malignant | **−** |
| D9 | 36 | M | 67 | Lung | Squamous cell carcinoma | 2 | II | T2N1M0 | Malignant | **−** |
| E1 | 37 | F | 64 | Lung | Adenocarcinoma | 2 | I | T2N0M0 | Malignant | **−** |
| E2 | 38 | F | 57 | Esophagus | Squamous cell carcinoma | 2 | IIa | T2N0M0 | Malignant | **−** |
| E3 | 39 | F | 67 | Esophagus | Adenocarcinoma | 3 | III | T4N0M0 | Malignant | + |
| E4 | 40 | F | 73 | Stomach | Mucinous adenocarcinoma | 3 | II | T2N1M0 | Malignant | **−** |
| E5 | 41 | M | 50 | Colon | Adenocarcinoma | 2 | II | T3N0M0 | Malignant | 2+ |
| E6 | 42 | F | 71 | Intestine | Intermediate grade malignant interstitialoma | – | IIb | T2bN0M0 | Malignant | **−** |
| E7 | 43 | M | 74 | Colon | Adenocarcinoma | 2 | II | T3N0M0 | Malignant | 2+ |
| E8 | 44 | M | 54 | Abdominal cavity | Malignant interstitialoma | – | IIb | T2N0M0 | Malignant | **−** |
| E9 | 45 | F | 50 | Rectum | Adenocarcinoma | 2 | II | T3N1M0 | Malignant | + |
| F1 | 46 | M | 32 | Rectum | Interstitialoma | – | IIa | T1bN0M0 | Malignant | **−** |
| F2 | 47 | M | 61 | Liver | Hepatocellular carcinoma | 2 | II | T2N0M0 | Malignant | **−** |
| F3 | 48 | F | 17 | Liver | Hepatoblastoma | – | – | – | Malignant | **−** |
| F4 | 49 | M | 58 | Kidney | Clear cell carcinoma | 1 | II | T2N0M0 | Malignant | **−** |
| F5 | 50 | M | 76 | Prostate | Adenocarcinoma (carcinoma sparse) | – | IV | T3N1M1b | Malignant | **−** |
| F6 | 51 | M | 72 | Prostate | Adenocarcinoma (Gleason grade: 4 Gleason score: 4+5) | 4 | IV | T2N0M1 | Malignant | **−** |
| F7 | 52 | F | 45 | Uterus | Leiomyoma | – | – | – | Benign | **−** |
| F8 | 53 | F | 51 | Uterus | Endometrial adenocarcinoma | 3 | Ic | T1cN0M0 | Malignant | **−** |
| F9 | 54 | F | 39 | Uterus | Clear cell carcinoma | – | IIb | T2bN0M0 | Malignant | **−** |
| G1 | 55 | F | 36 | Uterine cervix | Squamous cell carcinoma | 2 | Ib | T1bN0M0 | Malignant | **−** |
| G2 | 56 | F | 35 | Uterine cervix | Squamous cell carcinoma | 2–3 | Ia | T1a2N0M0 | Malignant | + |
| G3 | 57 | F | 20 | Striated muscle | Embryonal rhabdomyosarcoma of left leg | – | Ia | T1aN0M0 | Malignant | **−** |
| G4 | 58 | F | 70 | Rectum | Malignant melanoma of rectum | – | II | T4N0M0 | Malignant | **−** |
| G5 | 59 | F | 64 | Skin | Basal cell carcinoma of head | – | II | T2N0M0 | Malignant | **−** |
| G6 | 60 | M | 46 | Skin | Squamous cell carcinoma of left chest wall | 2 | II | T3N0M0 | Malignant | **−** |
| G7 | 61 | M | 59 | Soft tissue | Neurofibroma of left waist | – | – | – | Benign | **−** |
| G8 | 62 | F | 3 | Retroperitoneum | Ganglioneuroblastoma | – | – | – | Malignant | **−** |
| G9 | 63 | M | 48 | Abdominal cavity | Malignant mesothelioma | – | – | – | Malignant | **−** |
| H1 | 64 | F | 48 | Lymph node | Diffuse large B-cell lymphoma of right oxter | – | – | – | Malignant | **−** |
| H2 | 65 | F | 50 | Lymph node | Diffuse B cell lymphoma of right thigh | – | – | – | Malignant | **−** |
| H3 | 66 | M | 53 | Lymph node | Hodgkin's lymphoma over right clavicle | – | – | – | Malignant | **−** |
| H4 | 67 | F | 68 | Lymph node | Diffuse malignant lymphoma of mandible | – | – | – | Malignant | **−** |
| H5 | 68 | F | 72 | Bladder | Transitional cell carcinoma | 3 | II | T2N0M0 | Malignant | 2+ |
| H6 | 69 | M | 62 | Bladder | Leiomyosarcoma | – | IIb | T2N0M0 | Malignant | **−** |
| H7 | 70 | F | 7 | Bone | Osteosarcoma of right femur inferior extremity | – | IIIb | T2N0M0 | Malignant | **−** |
| H8 | 71 | F | 48 | Retroperitoneum | Spindle cell rhabdomyosarcoma of retroperitoneum | – | IIb | T2N0M0 | Malignant | **−** |
| H9 | 72 | F | 60 | Smooth muscle | Intermediate grade malignant leiomyosarcoma of left buttock | – | IIIb | T2bN0M0 | Malignant | **−** |
| – | – | M | 58 | Skin | Malignant melanoma (tissue marker) | – |  |  | Malignant | **−** |

^1^Grade 1–3 is equivalent to well-differentiated, moderately differentiated, and poorly differentiated, respectively. Grade 4 (for certain tumors) means that features are not sufficiently unique to distinguish the cells from undifferentiated cancers that occur in other organs.

^2^NAT: normal adjacent tissue.
