# Supplementary figures and images for: Blockade of phospholipid scramblase 1 with its N-terminal domain antibody reduces tumorigenesis of colorectal carcinomas in vitro and in vivo
Source: J Transl Med. 2012 Dec 24;10:254. doi: 10.1186/1479-5876-10-254 (PMC3551821; doi:10.1186/1479-5876-10-254)

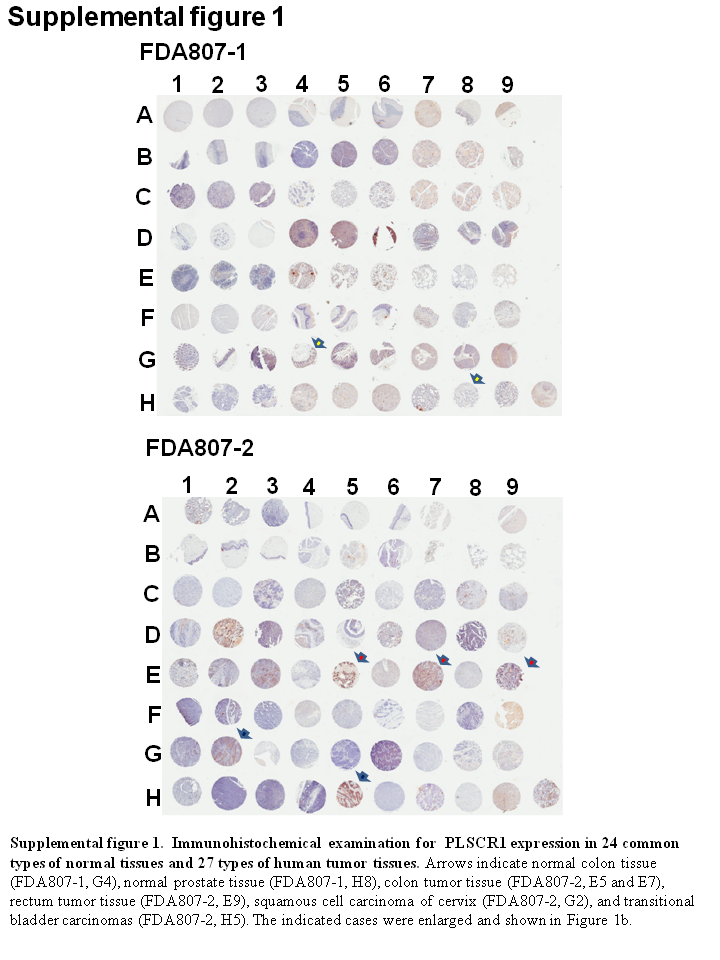

Supplement: Additional file 2 — Figure S1. Immunohistochemical examination for PLSCR1 expression in 24 common types of normal tissues and 27 types of human tumor tissues. [file 1479-5876-10-254-S2.tiff]

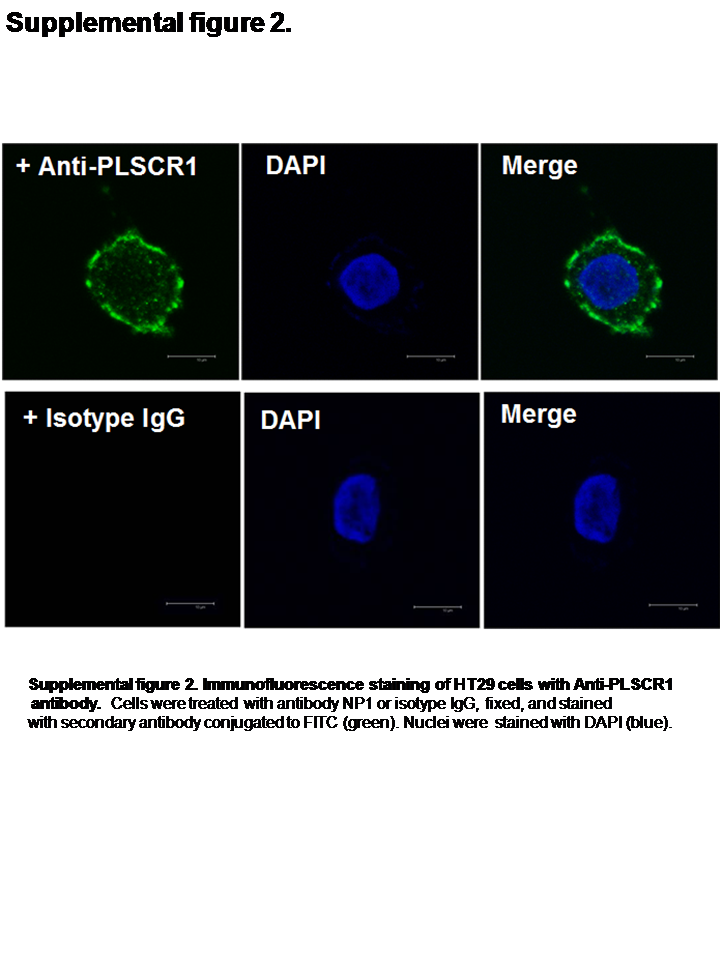

Supplement: Additional file 3 — Figure S2. Immunofluorescence staining of HT29 cells with Anti-PLSCR1 antibody. [file 1479-5876-10-254-S3.tiff]

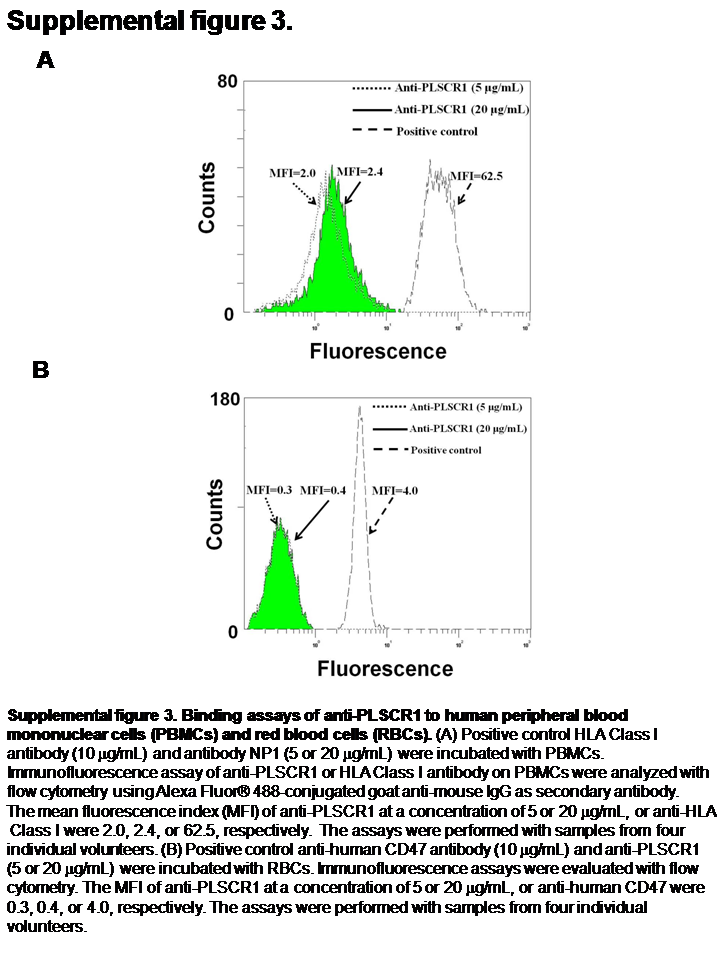

Supplement: Additional file 4 — Figure S3. Binding assays of anti-PLSCR1 to human peripheral blood mononuclear cells (PBMCs) and red blood cells (RBCs). [file 1479-5876-10-254-S4.tiff]

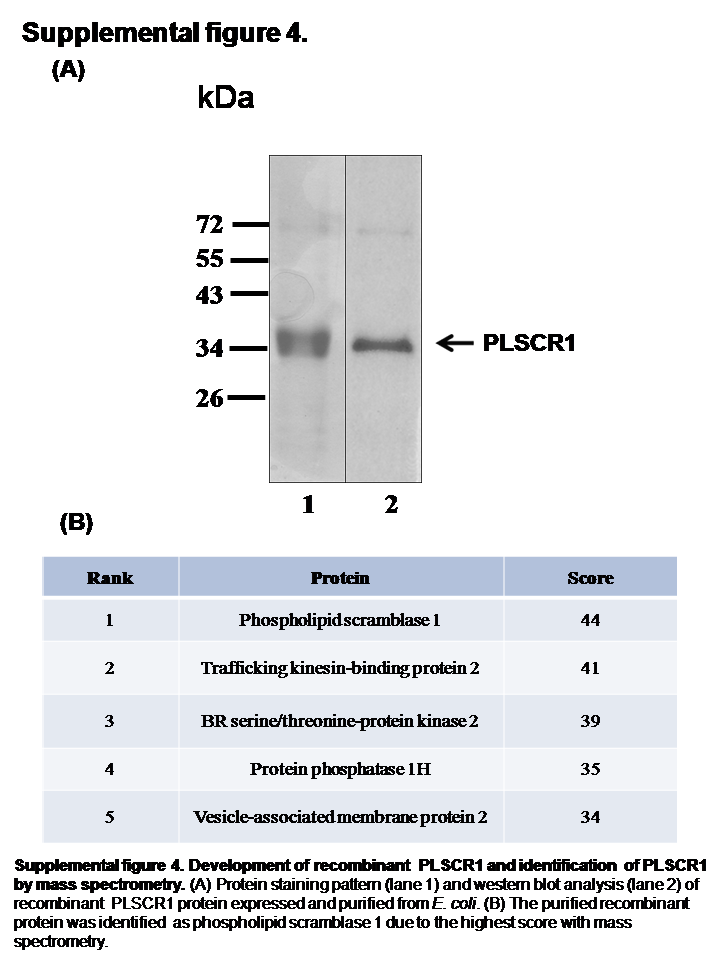

Supplement: Additional file 5 — Figure S4. Development of recombinant PLSCR1 and identification of PLSCR1 by mass spectrometry. [file 1479-5876-10-254-S5.tiff]
