# Supplementary material for: Blockade of phospholipid scramblase 1 with its N-terminal domain antibody reduces tumorigenesis of colorectal carcinomas in vitro and in vivo
Source: J Transl Med. 2012 Dec 24;10:254. doi: 10.1186/1479-5876-10-254 (PMC3551821; doi:10.1186/1479-5876-10-254)
Supplement: Additional file 6 — Preparation of recombinant PLSCR1 protein. [file 1479-5876-10-254-S6.doc]

**Preparation of recombinant PLSCR1 protein**

Total RNA was prepared from colorectal adenocarcinoma HT29 cells using the TRIzol^TM^ reagent（Invitrogen, Carlsbad, CA）. First-strand cDNA was synthesized in a total volume of 20μL using 2 μg of total RNA, 500 ng of oligo-dT primers（Invitrogen, Carlsbad, CA）, and SuperScript II reverse transcriptase（Invitrogen, Carlsbad, CA） acoording to the manufacturer’s protocol. The sequences of primers were as follows: Rplscr1, 5’ -AGGCATATGGACAAACAAAACTCACAG-3’’(sense), and 5’-GTTGGATCCCTACCACACTCCTGATT -3’(antisense). The PCR cycling conditions consisted of a single incubation step at 95℃ for 5 min, followed by touch-down PCR cycles of 1 min at 95℃, 1 min at 66℃ and 2 min at 72℃. The primer annealing temperature decrease 0.5℃ per program cycle till to 54℃. The reaction products were subcloned into pET14b (Novagen, Darmstadt, Germany) to produce a fusion protein containing N-terminal His_6_ tag. The recombinant proteins expressed in *Escherichia coli* BL21 (DE3)pLysS were purified using a HiTrap Chelating column (Pharmacia, Sunnyvale, CA). Expression of the recombinant PLSCR1 was examined using SDS-PAGE analysis followed by Coomassie blue staining to verify that the expression products had the expected molecular mass. In addition, the identity of the recombinant antigen fragments was confirmed by immunoblotting using a mouse monoclonal antibody against the human PLSCR1 (Invitrogen, Carlsbad, CA).
